# Cytogenetic analysis in the neotropical fish *Astyanax goyacensis* Eigenmann, 1908 (Characidae, incertae sedis): karyotype description and occurrence of B microchromosomes

**DOI:** 10.1186/1755-8166-6-48

**Published:** 2013-11-05

**Authors:** Luana Pereira dos Santos, Jonathan Pena Castro, Carine Mendonça Francisco, Marcelo Ricardo Vicari, Mara Cristina de Almeida, Leonardo Gusso Goll, Sandra Morelli, Roberto Ferreira Artoni

**Affiliations:** 1 Instituto de Genética e Bioquímica, Universidade Federal de Uberlândia, Av. Pará, 1720, 38400-902 Uberlândia, MG, Brazil; 2 Departamento de Biologia Estrutural, Molecular e Genética, Programa de Pós Graduação em Biologia Evolutiva, Universidade Estadual de Ponta Grossa, Avenida Carlos Cavalcanti 4748, 84030-900 Ponta Grossa, PR, Brazil

**Keywords:** Supernumerary chromosome, Synteny, 18S rDNA, Microdissection

## Abstract

**Background:**

B chromosomes, also known as supernumerary or accessory chromosomes, are additional chromosomes over the standard complement found in various groups of plants and animals. We investigated the presence of, and characterized, supernumerary microchromosomes in *Astyanax goyacensis* using classical and molecular cytogenetic methods.

**Findings:**

Three specimens possessed 2n = 50 chromosomes (8m + 26sm + 8st + 8a), and two specimens contained 1 to 9 additional B microchromosomes varying intra- and inter-individually. Chromosome painting with a B chromosome-specific probe yielded signals for several B microchromosomes, with one exhibiting no markings. Acrocentric chromosomes of the standard complement were also painted. Fluorescence *in situ* hybridization (FISH) using ribosomal probes located two chromosome pairs carrying 18S rDNA marked on the short arm, and one pair carrying 5S rDNA with pericentromeric markings. One chromosome was observed in synteny with 18S cistrons.

**Conclusion:**

These data contribute to knowledge of the karyotype evolution, the origin of B chromosomes, and to an understanding of the functionality of rDNA.

## Background

The B chromosomes, also known as supernumerary or accessory chromosomes, are additional chromosomes over the standard complement and are found in various groups of plants and animals. They are frequently characterized as heterochromatic and do not follow Mendelian principles of segregation [1]. The occurrence of these elements in *Astyanax* has been analyzed and is one of the most striking cytogenetic features of these fishes. Their occurrence has been well studied in *A. scabripinnis*[2] and noted in other species [3].

Neotropical fishes are extremely diverse, and *Astyanax* is among those with the greatest variety, with more than 100 known species [4]. This diversity hinders the identification and taxonomic description of the group, and these fishes have been categorized as *incertae sedis* among the Characidae [5]. Species complexes are common in *Astyanax* and are reported for *A. scabripinnis*[6], *A. altiparanae*[7], and *A. fasciatus*[8]. Karyotype variability follows the systematic diversity of *Astyanax*.

Garutti and Langeani [9] recently re-described *Astyanax goyacensis*, highlighting that *A. goyacensis* is a little studied species belonging to the *A. bimaculatus* species complex. The current study describes the karyotype of *A. goyacensis*, in which the presence of supernumerary microchromosomes was identified and characterized using classical and molecular cytogenetic methods.

## Results and discussion

*Astyanax goyacensis* contained 2n = 50 chromosomes and a heterochromatic B microchromosome ranging from 1 to 9. These B microchromosomes share sequences with acrocentric chromosomes of the standard complement.

**Figure 1:**
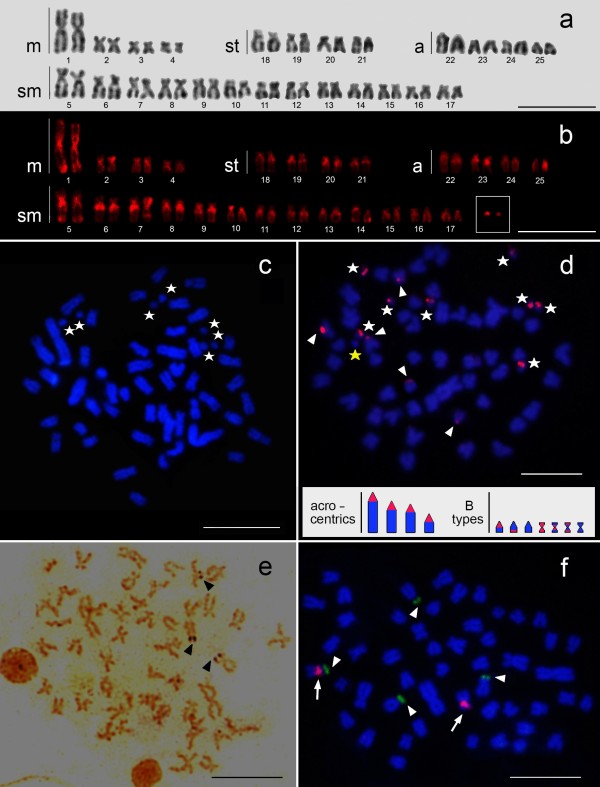
***Astyanax goyacensis *chromosomes showing (a) Giemsa-stained standard karyotype; (b) C-banding karyotype; (c) the mitotic metaphases (DAPI staining) show the B chromosomes (stars); (d) FISH with B chromosome-specific probe (arrowheads for autosomes and stars for the B microchromosome).** In detail a haploid idiogram of the acrocentric chromosomes and the types of B microchromosomes. Red markings indicate the location of the B chromosome-specific probe; **(e)** Ag-NORs bearing chromosomes (arrowheads); **(f)** FISH with 18S rDNA (green, arrowheads) and 5S rDNA (red, arrow) probes. The scale bars: 10 μm.

Cytogenetic studies have been conducted on several *Astyanax* species. In the present study, the karyotype of *A. goyacensis*, a little studied species with distribution throughout the Tocantins River basin, is described for the first time [9]. The conserved diploid chromosome number (2n = 50) is an established characteristic of *Astyanax* belonging to the *bimaculatus* group [10] and is confirmed here for *A. goyacensis*. An asymmetric karyotype characterized by the presence of different morphological types, as often observed in the genus, is described, with a karyotype formula comprising 8m, 26sm, 8st, and 8a (Figure 1a).

Two of the analyzed specimens of *A. goyacensis* possessed from 1 to 9 additional B microchromosomes and vary intra- and inter-individually (Figure 1b, c, d). The presence of additional chromosomes in *Astyanax* has been reported, particularly in *A. scabripinnis*[2]. As a rule, these chromosomes are large and are sometimes compared to the first pair of A chromosomes [11]. However, cases of *Astyanax* containing the B microchromosome are rare [12-14].

B chromosomes are, in most cases, heterochromatic, which is their most frequently cited cytogenetic characteristic [1]. The B microchromosomes identified in *A. goyacensis* were frequently heterochromatic, although supernumerary euchromatic examples were also noted (Figure 1b). Chromosome painting with a B chromosome-specific probe produced a signal in most B chromosomes along with hybridization signals on chromosomes of the standard complement (Figure 1d). The results suggest that the B chromosomes originated from chromosomes of the standard complement in addition to possessing heterochromatins similar to A chromosomes. Thus, two pairs of acrocentric chromosomes appear to be the most parsimonious candidates (Figure 1d). The development of B chromosomes from A chromosomes has been detected in fishes such as *Prochilodus lineatus*[8,15] and in the *Astyanax* genus [16,17].

The variable number of intra- and inter-individual B chromosomes indicates mitotic instability, with their accumulation reflecting an initial parasitic state of establishment and fixation in *A. goyacensis*. According to Camacho et al. [1], the accumulation of B chromosomes per cell and the variation in number of those chromosomes at the individual and population levels are characteristic of a parasitic state that precedes a more stable state in population oscillatory phenomena.

Multiple nucleolar organizer regions are common among *Astyanax*[18] and were evident in this study regardless of their activity (Figure 1e). Fluorescence *in situ* hybridization with ribosomal probes identified the location of two chromosome pairs carrying 18S rDNA marked on the short arm and one pair carrying 5S rDNA with pericentromeric markings; one chromosome was visualized in synteny with 18S cistrons (Figure 1f).

The most conserved pattern of the 5S rDNA location in fishes is the interstitial region of a single chromosome pair [19]. A similar finding is expected for *Astyanax*[20], although some species exhibit multiple markings in some chromosome regions [21]. In *A. goyacensis*, only one chromosome exhibits the syntenic marking of 18S and 5S rDNAs, while the homologue exhibits only 5S rDNA marking. A transposition event may have led to this synteny condition. Reciprocal translocation of the short arm and transposon-mediated 18S invasion are hypotheses that should be tested.

## Conclusion

*A. goyacensis,* assessed for its chromosomal complement for the first time, exhibited karyotype features that are shared with other *Astyanax* of the *bimaculatus* group. This provides an additional study model in evolutionary cytogenetics, given the presence of supernumerary microchromosomes and a polymorphic location of the ribosomal DNA multigene families.

## Methods

A cytogenetic survey was conducted on 5 specimens (3 female and 2 male) of *A. goyacensis* collected from the Taboquinha stream, a tributary of the Tocantins River basin located in the town of Monte do Carmo in the state of Tocantins, Brazil (S 10° 47′ 11.12″ and W 48° 13′ 16.57″). The specimens were euthanized using the benzocaine, identified, and deposited in the Laboratory of Systematic Zoology, Sagrado Coração University, Bauru-SP, under voucher number CZUSC 00102. The study was performed with permission from the Chico Mendes Institute for Biodiversity Conservation [Instituto Chico Mendes de Conservação da Biodiversidade (ICMBio)] through the authentication code 43852933. Mitotic chromosomes were obtained from cells of the anterior kidney using the air-drying procedure [22]. The chromosome preparations were submitted to conventional Giemsa staining, C banding [23] and Ag-NORs [24] to determine the diploid number, chromosome morphology, distribution of heterochromatin, and presence of B chromosomes. The chromosome morphology was determined using arm ratios [25], and chromosomes were classified as metacentrics (m), submetacentrics (sm), subtelocentrics (st), and acrocentrics (a).

The microchromosomes B (B-probes) were obtained via microdissection of mitotic metaphase cells from the single specimen that carried only a B microchromosome, using an inverted IX51 microscope (Olympus) equipped with a Transferman mechanical micromanipulator (Eppendorf). The B chromosomes were submitted to a degenerated oligonucleotide primer–polymerase chain reaction (DOP-PCR), following the procedure described by [26] with slight modifications [27]. A second amplification reaction was performed using 100 ng of the first PCR product in a final volume of 50 μL. The reaction solution consisted buffer (200 mM Tris, pH 8.4 and 500 mM KCl), 1 × *Taq* DNA polymerase (Invitrogen), 2 mM MgCl_2_, 400 μM dNTP, 2 μM DOP primer 5′-CCGACTCGAGNNNNNNATGTGG-3′, and 2 U *Taq* DNA polymerase. The amplification was performed in a thermocycler (Biocycler) using the following program: three minutes at 94°C; 35 cycles of 90 sec at 90°C, 90 sec at 52°C, and 90 sec at 72°C; and a post-cycling extension of 5 min at 72°C.

The B-probe was labeled through the nick translation procedure using digoxigenin-16-dUTP (DIG-Nick Translation Mix, Roche Applied Science). The PCR mixture consisted of 1 × *Taq* DNA polymerase buffer (Invitrogen); 2 mM MgCl_2_; 40 μM dTTP, dGTP and dCTP; 20 μM dATTP; 20 μM biotin-14-dATP; 2 μM DOP primer; and 2 U *Taq* DNA polymerase.

The ribosomal probes (18S rDNA and 5S rDNA) were mapped via FISH on the *A. goyacensis* chromosomes. The 18S rDNA probe was labeled with biotin through nick translation using biotin-16-dUTP (Biotin-Nick Translation Mix, Roche Applied Science), and the 5S rDNA probes were labeled through nick translation using digoxigenin-16-dUTP (DIG-Nick Translation Mix, Roche Applied Science).

Chromosome painting with the B-probe and FISH were performed under conditions of high stringency (2.5 ng/μL probe, 50% formamide, 2× SSC, 10% dextran sulfate) following the general procedure described by [28]. Signal detection was conducted with anti-digoxigenin-rhodamine (Roche) and Avidin-FITC (Sigma) antibodies. The chromosomes were counterstained with DAPI (0.2 μg/mL) in VECTASHIELD^®^ mounting medium (Vector) and analyzed using an epifluorescence microscope (Olympus BX41) coupled with a DP71 imaging system (Olympus).

## Abbreviations

Ag-NORs: Silver nitrate staining; 2n: Diploid number; m: Metacentric chromosome; sm: Submetacentric chromosome; st: Subtelocentric chromosome; a: Acrocentric chromosome; FISH: Fluorescence in situ hybridization; rDNA: Ribosomal DNA; PCR: Polymerase chain reaction; DOP-PCR: Degenerated oligonucleotide primer–polymerase chain reaction.

## Competing interests

The authors declare that they have no competing interests.

## Authors’ contributions

LPS collected the animals, performed the cytogenetic studies and helped draft the Manuscript. JPC performed some cytogenetic preparations and production of probes. CMF helped to collect the animals and performed the cytogenetic studies. MRV MCA LGG helped perform some cytogenetic preparations. SM participated in its design and coordination. RFA supervised the experiments studies, drafted the manuscript and revised the final text. All authors read and approved the final manuscript.
